# An Improved Ambiguity-Free Method for Precise GNSS Positioning with Utilizing Single Frequency Receivers

**DOI:** 10.3390/s20030856

**Published:** 2020-02-06

**Authors:** Wenhao Yang, Yue Liu, Fanming Liu

**Affiliations:** College of Automation, Harbin Engineering University, Harbin 150001, Chinaliuyuehrb@outlook.com (Y.L.)

**Keywords:** differential precise GNSS positioning, ambiguity-free, MAFA, single-frequency receiver

## Abstract

The solution of carrier phase ambiguity is essential for precise global navigation satellite system (GNSS) positioning. Methods of searching in the coordinate domain show their advantage over the methods based on ambiguity fixing, for example, immune to cycle slips, far fewer epochs taken for obtaining the precise solution. However, there are still some drawbacks via using the Ambiguity Function Method (AFM), such as low computation efficiency and the existence of a false global optimum. The false global optimum is a situation where the Least Square (LS) criterion achieves minimum in another place than the point of the actual position, which restricts the application of this method to single-frequency receivers. The numerical search approach derived in this paper is based on the Modified Ambiguity Function Approach (MAFA). It focuses on eliminating the false optimum solution and reducing the computation load by utilizing single-frequency receivers without solving the ambiguity fixing problem. An improved segmented simulated annealing method is used to decrease the computation load while the Kernel Density Estimator (KDE) method is used to filter out the false optimum candidates. Static experiments were carried out to evaluate the performance of the new approach. It is shown that a precise result can be obtained by handling two epochs of data with *z* coordinate fixed to the referenced value. Meanwhile, the new approach can achieve a millimeter level of position accuracy after dealing with nineteen epochs of observations data when searching in x,y,z domain. The new approach shows its robustness even if the search region is broad, and the prior position is several meters away from the referenced value.

## 1. Introduction

the global navigation satellite system (GNSS) differential positioning method is is widely used nowadays owing to its advantage of the effective cancellation of common error sources, for example, ionosphere and troposphere errors [1,2,3,4]. The carrier phase is the most precise measurement available for precise GNSS positioning, but the integer ambiguities of the carrier phase cycles should be determined [5,6]. Different approaches to this problem have appeared over the past years, one category of algorithms searches for the final solution in the ambiguity domain. Well-known examples are integer least squares (ILS), integer bootstrapping (IB), and integer rounding (IR) [7]. The ILS estimator is the optimal solution in the Gaussian case, and the least-squares ambiguity decorrelation adjustment (LAMBDA) is today widely used method owing to its computation efficiency based on the decorrelation between estimated ambiguities [8]. However, the LAMBDA method needs a very high accuracy approximate position, and it is susceptible to cycle slips.

The second category of algorithms is searching in the position domain, and the representative algorithm is the ambiguity function method (AFM) [9]. It can achieve high precision positioning results without the need for accuracy of prior position and complicated ambiguity resolution [10,11,12]. However, AFM performance is easily affected by multipath and unmodeled errors [13,14]. It also has a problem in that there may be several maxima points; the AFM algorithm must filter out the wrong peak points in the search region in order to identify the optimal position. Another drawback of using the AFM is the low computation efficiency owing to the need for the searching step to be much less than the carrier phase wavelength; for example, 19 cm for the $L_{1}$ carrier phase and 24 cm for the $L_{2}$ carrier phase. Many methods have been proposed to solve these problems. Han et al. [15] described the techniques that improve the reliability of the AFM and significantly shorten the computation time necessary for the AFM by taking advantage of optimal dual-frequency observable combinations. Xin Li et al. [16] proposed a new approach for deformation monitoring of a super high-rise building. The approach is based on the AFM, and an improved particle swarm optimization (IPSO) algorithm is used to increase the computation speed. However, this method still needs relatively precise known coordinates and relatively small amplitude variations over a short span. S.Cellmer et al. [17] presented a meaningful modified ambiguity function approach (MAFA) for GNSS precise positioning. It is a method of carrier phase processing based on the least-squares adjustment algorithm and specific properties of AFM. Thus it ensures the condition of parameter “integerness” without the need for the stage of the integer search. However, a high accuracy approximate position is needed. Further investigations of the MAFA method are mainly about the search accuracy and efficiency. In Reference [18], the integer decorrelation procedure was used to improve the computation efficiency of MAFA. A new search procedure was proposed in Reference [19] to improve the MAFA method to obtain the correct solution even in the case when a priori position is a few meters away from the actual position. The necessary condition for obtaining the correct solution in MAFA was shown in Reference [20], that is, the approximate position has to be inside the Voronoi cell of the “true” position. In Reference [21], a solution to minimize the size of the search cube in the MAFA method was presented. In Reference [22], the probabilistic aspect was taken into account, and the search region was formed with a certain confidence level. Above all, the AFM class methods indeed show their advantage over the LAMBDA method, which is immune to cycle slips, especially the MAFA method may give out the precise positioning result by using a single epoch of data even in the case of poor accuracy of approximate position. However, the decreasing of the computation load is mainly done by using the cascade adjustment with linear combinations of carrier phase observations. With the high price of the dual-frequency receivers, there is still a need for precise and fast GNSS positioning by using a single frequency receiver based on the AFM class method. Some related work can be found in References [23,24]. However, the existence of the false global optimum, which is a situation where the Least Square (LS) criterion achieves minimum in another place than the point of the actual position, is still a big challenge for utilizing the AFM class methods and the other methods based on the carrier phase data to achieve a precise positioning [25]. It is especially prominent for single frequency receivers to use the AFM class search method when the accuracy of the prior position is too poor, and the search region is too large.

In the present paper, a numerical search approach for precise GNSS positioning based on the MAFA method and single-frequency receivers is proposed. An improved segmented simulated annealing method is used to decrease the computation load of searching for the correct solution in the position domain. A Kernel Density Estimator (KDE) method is used to filter out the other false optimum candidates based on the multi-epochs search results and obtain the point with the correct position. For the sake of convenience, this new approach is called the Segmented Simulate Annealing Modified Ambiguity Function Approach (SSA-MAFA).

The remainder of the paper is organized as follows—in Section 2, the proposed algorithm is described in detail. Experimental and analyses are presented in Section 3. The paper ends with conclusion in Section 4.

## 2. Methodology

**Figure 1:**
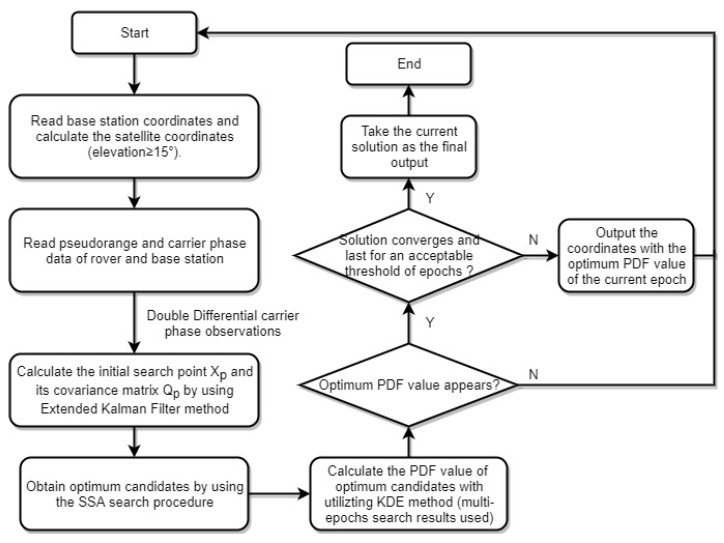
The whole procedure of the Segmented Simulate Annealing Modified Ambiguity Function Approach (SSA-MAFA) algorithm.

The new approach derived in this paper is based on the MAFA method. It focuses on eliminating the false optimum solution and reducing the computation load by utilizing single-frequency receivers without solving the ambiguity fixing problem. The whole procedure is shown in Figure 1.

### 2.1. MAFA Method

The Modified Ambiguity Function Approach (MAFA) is based on the least squares adjustment (LSA) algorithm. Although this method does not calculate the full-cycle ambiguities directly, the integer nature of ambiguities is ensured in the results, owing to the suitable constraints adopted in the function model [26].

Considering the foremost cause of error in the positioning system can be vanished by differencing, the double differential (DD) observation model is used in the method and formulated as:

(1) $$\Phi =\frac{1}{\lambda }\rho \left(X_{c}\right)+a+e,$$

where Φ, DD carrier phase measurement(in cycles); λ, signal wave length(in meters); *e*, measurement noise(in cycles); $\rho (X_{c})$, DD geometrical range of the correct receiver position; and *a*, DD integer number of cycles;

For simplicity, the term $\rho (X_{c})$ is represented as ρ in subsequent equations, Equation (1) can be rewritten as

(2) $$e=(\Phi -\frac{1}{\lambda }\rho )-a.$$

It is proved that the nominal accuracy of carrier phase measurement is about 0.01 cycles [27]. As a fact, the residual values can be much lower than half a cycle [28]. Considering the integer nature of the ambiguity parameter *a*, Equation (2) can be rewritten in the following form:

(3) $$e=\left(\Phi -\frac{1}{\lambda }\rho \right)-round\left(\Phi -\frac{1}{\lambda }\rho \right),$$

where round(.) is a function of rounding to the nearest integer. A Taylor series expansion of the system of equations can be represented as:

(4) $$e=\Delta -\frac{1}{\lambda }Bb,$$

with

(5) $$\Delta =\left[\begin{array}{c} \delta_{1}\\ \delta_{2}\\ \vdots \\ \delta_{n} \end{array}\right],$$

(6) $$\delta_{n}=\left(\Phi_{n}-\frac{1}{\lambda }\rho_{n}\left(X_{0}\right)\right)-round\left(\Phi_{n}-\frac{1}{\lambda }\rho_{n}\left(X_{0}\right)\right),$$

(7) $$B=\left[\begin{array}{ccc} \frac{\partial \rho_{1}}{\partial x} & \frac{\partial \rho_{1}}{\partial y} & \frac{\partial \rho_{1}}{\partial z}\\ \frac{\partial \rho_{2}}{\partial x} & \frac{\partial \rho_{2}}{\partial y} & \frac{\partial \rho_{2}}{\partial z}\\ \vdots & \vdots & \vdots \\ \frac{\partial \rho_{n}}{\partial x} & \frac{\partial \rho_{n}}{\partial y} & \frac{\partial \rho_{n}}{\partial z} \end{array}\right],$$

where e, error vector (n×1); *b*, parameter vector (increments to a prior coordinates vector $x_{0}$); *B*, design matrix (n×3); Δ, misclosure vector (n×1); $x_{0}$, approximate coordinate of the receiver; $\rho (X_{0})$, DD approximate geometrical range (DD geometric distance vector computed using a prior position and satellite coordinates); *n*, number of DD observations.

Thus adjustment problem can be formulated as:

(8) $$\arg\min_{b}(MAFV=e^{T}We),$$

with *W* standing for the weight matrix, *W* can be formed as:

(9) $$W={\left(DRD^{T}\right)}^{-1},$$

with

(10) $$D=\frac{1}{\lambda }{\left[\begin{array}{ccccc} 1 & -1 & 0 & \cdots & 0\\ 1 & 0 & -1 & \cdots & 0\\ \vdots & \vdots & \vdots & \ddots & \vdots \\ 1 & 0 & 0 & \cdots & -1 \end{array}\right]}_{n\times n},$$

(11) $$R=\left[\begin{array}{cccc} \sigma_{1}^{2} & \; & \; & \;\\ \; & \sigma_{2}^{2} & \; & \;\\ \; & \; & \ddots & \;\\ \; & \; & \; & \sigma_{n}^{2} \end{array}\right],$$

where $\sigma_{n}$, standard deviation of phase-range measurement error of the *n* satellites. It can be calculated by the following formulas:

(12) $$\sigma_{n}^{2}=2\left(a^{2}+b^{2}/sin^{2}El^{n}+c^{2}\right)+d^{2},$$

where a,b,c, carrier phase error factor( in meters); El, the elevation angels of satellite *n*; *d*, satellite clock stability(sec/sec).

The solution of this problem is the following parameter vector:

(13) $$b=-\lambda {\left(B^{T}WB\right)}^{-1}B^{T}W\Delta .$$

The results show that although there is no ambiguity parameter in the adjustment model, the result still satisfies the mixed integer-real LS (MIRLS). Therefore, it is not susceptible to cycle slips, and it is impossible to fix the wrong ambiguities, allowing accurate and reliable baseline determination.

However, if the accuracy of the approximate position is poor, then the Equation (6) should change to:

(14) $$\delta_{n}=\left(\Phi_{n}-\frac{1}{\lambda }\rho_{n}\left(x_{0}\right)\right)-round\left(\Phi_{n}-\frac{1}{\lambda }\rho_{n}\left(x_{0}\right)\right)+a_{e}\left(n\right).$$

A different search strategy was proposed based on the integer nature of the vector $a_{e}\left(n\right)$. Generally, these strategies can be divided into 2 domains, namely searching in the ambiguity domain and in the position domain. Both strategies consist in testing the values of Equation (8). In Reference [20], it was proved that the optimal global solution could be obtained as long as the search candidates do not omit the Voronoi cell of the correct solution. The Voronoi cell is a pull-in region, which has a single local optimal value among the points in the region corresponding to a set of integer ambiguities [29]. However, if the search area is too large owing to the poor accuracy of the approximate position, the false global optimum appears if the condition(15) is met by a group of observations that have a determinative impact on the criterion (8).

(15) $$\Phi_{n}-\frac{1}{\lambda }\rho_{n}\left(x_{w}\right))-round\left(\Phi_{n}-\frac{1}{\lambda }\rho_{n}\left(x_{w}\right)\right)<\left(\Phi_{n}-\frac{1}{\lambda }\rho_{n}\left(x_{c}\right)\right)-round\left(\Phi_{n}-\frac{1}{\lambda }\rho_{n}\left(x_{c}\right)\right),$$

where $x_{w}$, the center of the wrong Voronoi cell; $x_{c}$, the center of the correct Voronoi cell.

Cascade processing is usually used to speed up the search speed. Thus the false global optimum is hardly searched out owing to the large wavelength of wide lane combination. However, there is still a great chance to encounter the wrong global optimal solution via using the single-frequency receivers, which is shown in the experimental section. Therefore, the MAFA method is very likely to fail if the accuracy of the prior position is poor, and the search region is too large.

### 2.2. Method for Improving the Search Efficiency of MAFA

Another concern is the search efficiency of the MAFA. It was proved in Reference [22] that the optimal distance between the closest points in the search grid could be half of the wavelength. However, the point by point grid search method is still very low efficiency, considering the accuracy of prior position is usually about 1–2 m, which severely limits the application of MAFA by using the single-frequency receivers. In References [23], an adapted mixed half-stochastic, half-deterministic optimization algorithm based on the traditional simulated annealing method (SA) was proposed, which dramatically reduces the computational burden. However, this method is still affected by the false global optimum if the accuracy of the prior position is poor. Therefore, Segmented Simulated Annealing (SSA) method is introduced.

#### 2.2.1. Simulate Annealing Method Review

The traditional Simulated Annealing method (SA) is derived from the study of the formation process of a crystalline network. Three key features have to be implemented, namely the thermal agitation of molecules, the cooling scheme, and the acceptance criteria. At first, an initial temperature $T_{0}$, cooling coefficient *d*, termination temperature $T_{d}$ are determined. Among them, $T_{0}$ is a relatively large number, *d* is a number very close to 1, $T_{d}$ is a positive number close to 0. The current temperature is defined as $T_{0}$ at the beginning, and then the probability of state transition (modifying the optimal solution or setting the initial state for the next transition) is as follows:

(16) $$P\left(\Delta E\right)=\left\{\begin{array}{cc} 1 & new\;state\;is\;better\\ e^{\frac{-\Delta E}{T}} & new\;state\;is\;worse \end{array}\right.$$

where ΔE, the energy (value) difference between the known state and new state (obtained by adding Gaussian random to the known state). After the state transition, temperature *T* can be updated by:

(17) $$T_{k}=d\cdot T_{k-1}.$$

Repeat the state transition until $T_{k}$<$T_{d}$, the current optimal solution is the final optimal solution. For better illustration of the proposed method, the terminology was changed to match the problem in the subsequent sections: $T_{0}$: initial standard jump amplitude instead of initial temperature; $T_{d}$: final standard jump amplitude instead of termination temperature; *d*: decreasing coefficient of standard jump amplitude instead of the cooling coefficient.

#### 2.2.2. Segmented Simulated Annealing (SSA)

The traditional SA method is a metaheuristic to approximate global optimization in an ample search space for an optimization problem [30]. It has been proved that the success probability of this method for searching the global optimum approaches one [31]. However, the existence of the false global optimum makes the SA method fail to give out the correct solution. Considering the condition that gives out the correct solution is that the search point falls into the correct Voronoi cell. Even though the existence of a false global optimum, we could still find out the correct solution if the search points of SA can fall into the right cell, namely the local optimum. Therefore, if the changing states of the global optimum are all saved while the decreasing of the standard jump amplitude, the final result can still obtain the correct solution.

**Figure 2:**
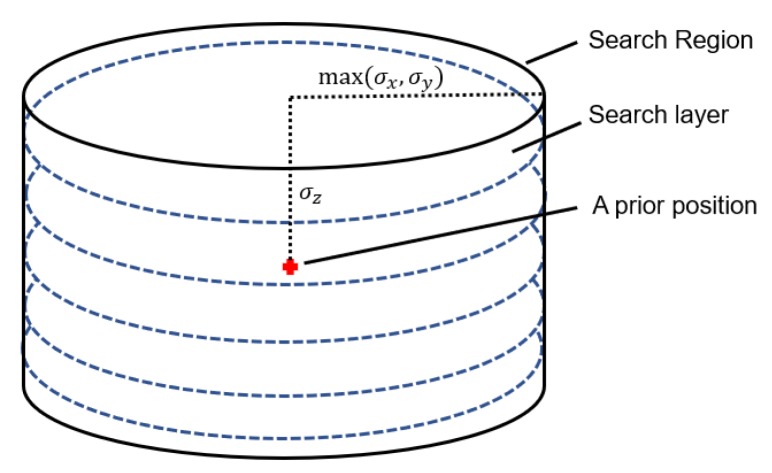
Illustration of the search region around the prior position and the set up of the search layers, where $\sigma_{x},\sigma_{y},\sigma_{z}$ is the standard deviation of the a prior position.

In order to make the search points fall into the correct cell with the highest probability, the search region can be divided into several search layers by dividing the search length in *Z* direction. The search cube can be chosen as a cylinder, and structured base on the coordinate vector of the a priori position $X_{0}$ and its covariance matrix $Q_{X_{0}}$, where $X_{0}$ can be obtained by using some traditional method, for example, Extended Kalman Filter. The distance between different search layers can be half of the signal wavelength [22], shown in Figure 2.

**Figure 3:**
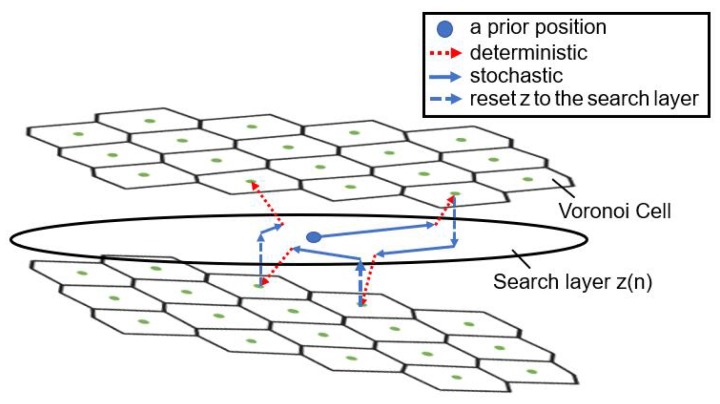
Illustration of search procedure on a particular search layer.

The new search method is called the Segmented Simulated Annealing (SSA) algorithm. As the illustration shown in Figure 3, the *Z* coordinate value is first settled to the corresponding search layer while maintaining the *X* and *Y* value of the *a* prior position. Equation (13) is solved to give out refined coordinates, which is called the deterministic step. Then Equation (8) is evaluated at the refined coordinates, and Equation (16) was used to decide whether to obtain this point as the global optimum or the origin search point for the next iteration. Then, the *Z* value of the search point is resettled to the value of corresponding search layer, and Gaussian random value is added to obtain a new search point, which is called the stochastic step. Repeat the above procedure until the searching procedure reaches its final standard jump amplitude and save all the optimum global solutions that appeared. It should be noticed that the probability of accepting the worse solution as the origin of the next iteration should be settled to a large number, for example, 0.3, in order to obtain a more number of optimum candidates. The whole procedure of the SSA algorithm is shown in Algorithm 1.
**Algorithm 1:** The procedure of the SSA algorithm
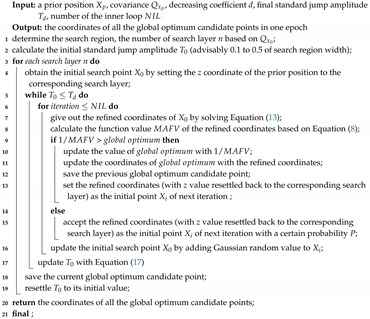


### 2.3. Kernel Density Estimation Method for Eliminating the False Optimum Candidates

There will be a large number of candidates of global optimum points via using the SSA method dealing with one epoch of observation data. Fortunately, the accuracy of the MAFA method is the millimeter level for the correct solution. Therefore, the Kernel Density Estimation (KDE) method can be used to eliminate the false global optimum points based on multi-epochs search results.

Kernel density estimation is a fundamental data smoothing problem where inferences about the population are made, based on a finite data sample [32], shown as follows:

(18) $$\hat{f_{h}}\left(x\right)=\frac{1}{nh}\sum_{i=1}^{n}K(\frac{x-x_{i}}{h}),$$

where *K*, the kernel, a non-negative function; *h*, a smoothing parameter called bandwidth; *n*, total number of data samples.

The value of bandwidth *h* reflects the overall flatness of the KDE curve, that is, the more significant the bandwidth, the smaller the proportion of observed data points in the final curve shape, the flatter the overall KDE curve. The smaller the bandwidth, the larger the proportion of observed data points in the final curve shape, the steeper the overall KDE curve. In the present approach, the triangular is used as the kernel function while the value of bandwidth *h* we choose as a tiny number, for example,1 cm. As a result, only the distance between optimum candidates in multi-epochs search results is no more than 1 cm can the more considerable Probability density function (PDF) value obtained.

## 3. Experiment and Analyses

**Figure 4:**
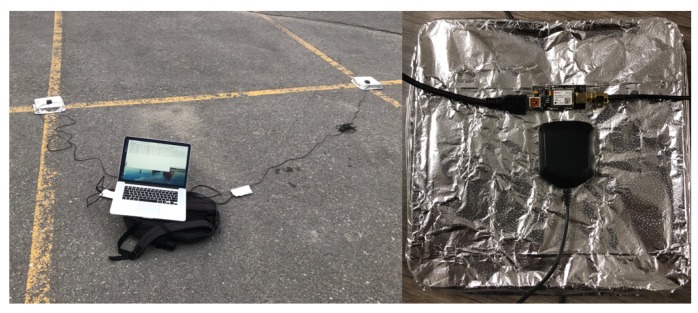
Experiment setup.

The static experiments were conducted at a parking lot at Queen’s University in Kingston, Ontario, Canada. Two u-Blox NEO-M8T single frequency receivers and low cost of antennas were used as the rover and base station respectively, only GPS, L1 pseudorange and carrier phase observations, were used. The precise positions of both receivers were prior obtained by using the RTKLIB software with the KNGS CORES station as the base station. The receivers were connected to a laptop computer via USB, with a sample rate settled at 5Hz. Data was post calculated using a PC with an Intel Core i7-6700T CPU, 16GB memory running Windows 10 operating system. The experiment setup is shown in Figure 4.

**Figure 5:**
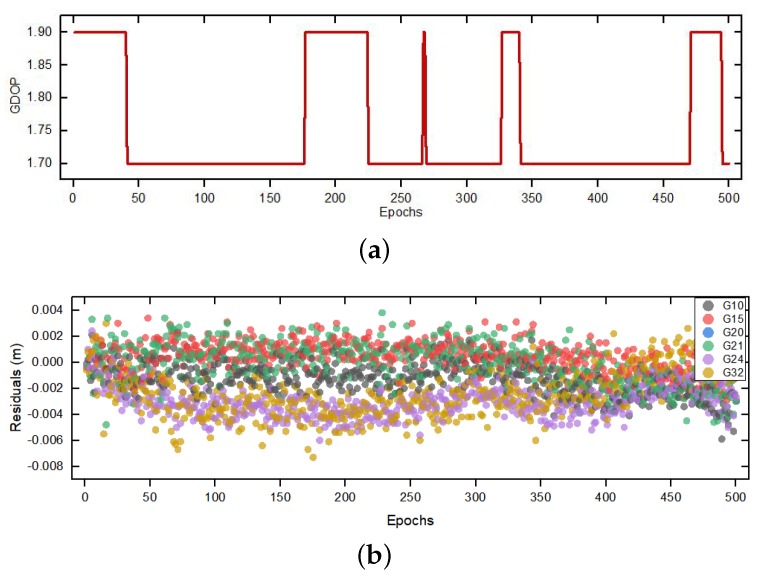
GDOP and Measurement residuals during the sampling periods (**a**) GDOP value. (**b**) The measurement residuals of carrier phase.

The experiment was carried out on 18 August 2019 00:06:26 (GPS Time), a total of 500 epochs of data were sampled, the GDOP and the residuals of carrier phase during sampling time are shown as Figure 5. The experiment is used to test the accuracy as well as the efficiency of our proposed SSA-MAFA ability to eliminate the false global optimum points.

### 3.1. Precision of SSA-MAFA

#### 3.1.1. Search with z Coordinate Fixed to Its Reference Value

**Figure 6:**
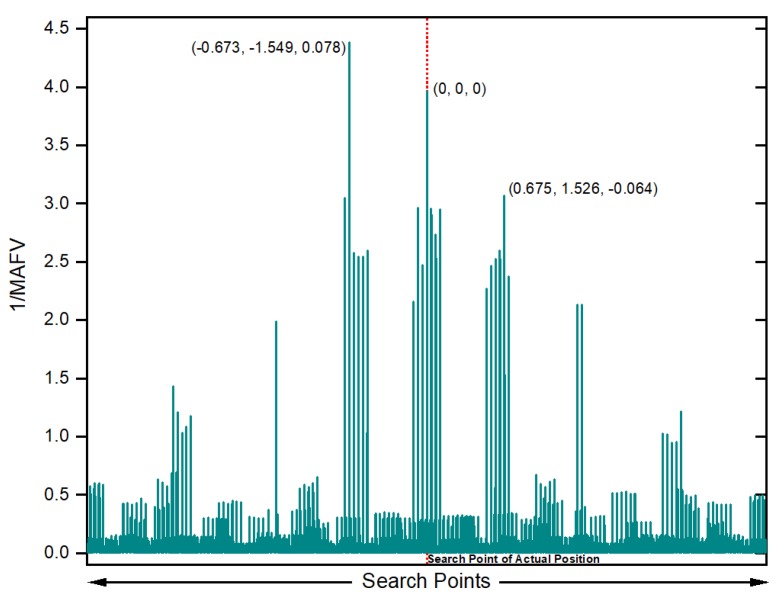
1/MAFV result for the search points with *z* coordinate fixed to its reference value, where the red dot line indicates the search point of actual position, the value in the brackets is the error of the solution point referenced to the actual position.

The SSA-MAFA method is an optimizing method that looks for the global optimum in each search layer and then use the kernel density method to eliminate the false global optimum points. Thus, the search accuracy and efficiency in the correct layer are of importance. The crucial feature of the MAFA method is restricting the set of possible solutions to only those points that take into account the integer nature of ambiguities. We first constructed the map of the function value in a design area of the correct search layer to find out the search accuracy of the MAFA method for the points that are or near the centers of Voronoi cells. For better illustration, the value of 1/MAFV is used as the function value. The *z* coordinate was fixed to referenced value while the search width, Δx, Δy settled to ±3 m around the correct point, the search step length was settled to 4 cm. Calculate the value of Equation (13) for each search point of the correct search layer and give out the 1/MAFV values for the solution points, the result is shown in Figure 6.

**Figure 7:**
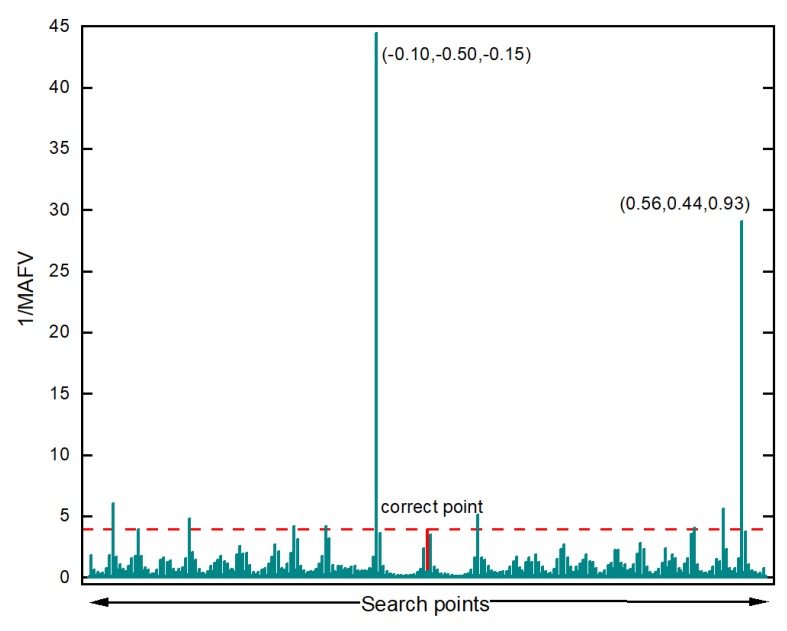
1/MAFV result in x,y,z domain: ±1 m around the referenced value.

The result shows that the solution value for the search point of the actual position is precisely the point itself, which means the point itself is the center of the correct Voronoi Cell. However, 1/MAFV value of the correct solution point is less than another point (−0.673, −1.549, 0.078), owing to the existence of *false global optimum situation*. Meanwhile, considering the error noise is not larger than 0.01 cycle of the wavelength, we can choose a threshold value of 1/MAFV when looking for the correct solution point, for example, 1/MAFV ≥ 1. Then we construct the map of 1/MAFV value with different *z* coordinate values, as shown in Figure 7.

It shows that in search of the 3D scene, the false optimum happens more often. There are 10 points that the value of 1/MAFV is more significant than the correct point. Nevertheless, the 1/MAFV value of coordinates(referenced to the correct position): (−0.1, −0.50, −0.15) and (0.56, 0.44, 0.93) are much larger than the correct point, which is 44.45 and 29.11 respectively, while the value of correct one is 3.96.

**Figure 8:**
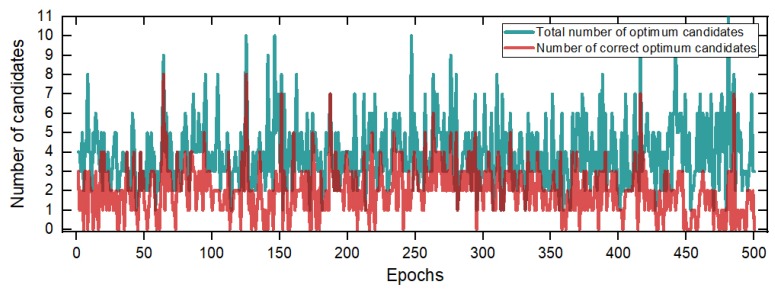
Number of optimum candidates with *z* coordinate fixed to referenced value.

Next, we test the SSA-MAFA method by setting the *z* coordinate to its reference value to verify its ability to find the global optimum on the correct search layer. The settlement of parameters in the test were as follows: d=0.9, NIL=50, $T_{0}=0.4\times 6$(0.4×*search region width*), $T_{d}=0.03$, the threshold for saving the optimum candidates was 1, a total of 500 epochs of data were tested. The initial point of each epoch used the result of the Extended Kalman Filter (EKF), while the *z* coordinate was settled to the referenced value manually. The number of optimum candidates in each epoch is shown in Figure 8.

**Figure 9:**
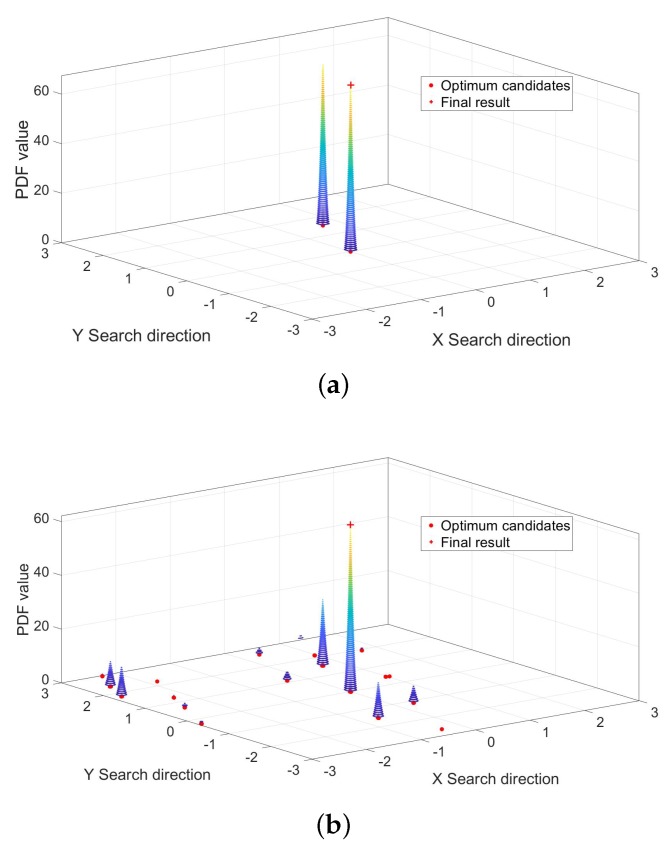
Probability density function (PDF) value of different Epochs. (**a**) PDF value of Epoch 2. (**b**) PDF value of Epoch 500.

**Figure 10:**
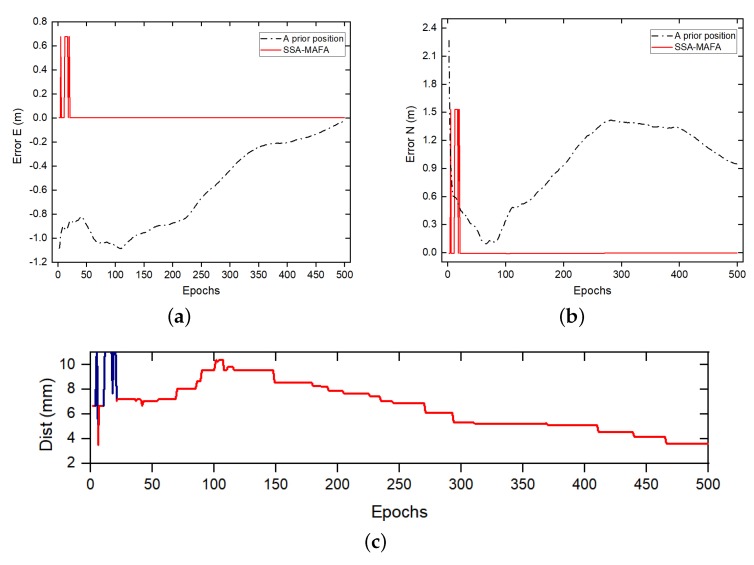
Error result of SSA-MAFA with *z* coordinate fixed to referenced value. (**a**) The error in East direction. (**b**) The error in North direction. (**c**) The error in East and North direction from epoch 1 to epoch 500, where Dist = $\sqrt{err_{E}^{2}+err_{N}^{2}}$, the blue line indicates the wrong solution.

The results demonstrate that 80% of epochs can the search algorithm find the correct optimum points. The reason for not finding the correct solution is mainly because the broader search region introduces a false global optimum, which makes the SSA-MAFA method failed to obtain the correct point. Meanwhile, there is the same accuracy of search points in one epoch. We filter the search result of one epoch data before we use the KDE method: if the search points are among a specific threshold range within each other, for example, 1 cm, remain only one as the optimum candidate point. The bandwidth *h* of KDE method was settled to 1 cm, the Probability density function (PDF) results of Epoch.2 and Epoch.500 are shown as Figure 9. The error results of the SSA-MAFA by using the KDE method with a *z* coordinate fixed to the referenced value are shown as Figure 10.

As is shown in Figure 9, the SSA-MAFA method can obtain a correct solution after handling with only two epochs of data even if the accuracy of the prior position is down to 2 m. There are nine epochs of solutions out of 500 failed to acquire the correct solution, shown as Figure 10. The reason can be found in Figure 8, the vast search region we set brings the false global optimum and leads to the failure of giving out the correct solution. It is worth noticing that 20% of epochs’ search results failed to obtain the correct optimum points, yet the final result is the correct solution. This is because the bandwidth *h* of the KDE method we used is small enough that the false optimum candidates of each epoch can not match with each other, which leads to a more considerable PDF value of the correct optimum candidate.

#### 3.1.2. Search in both x, y and z Domain

**Figure 11:**
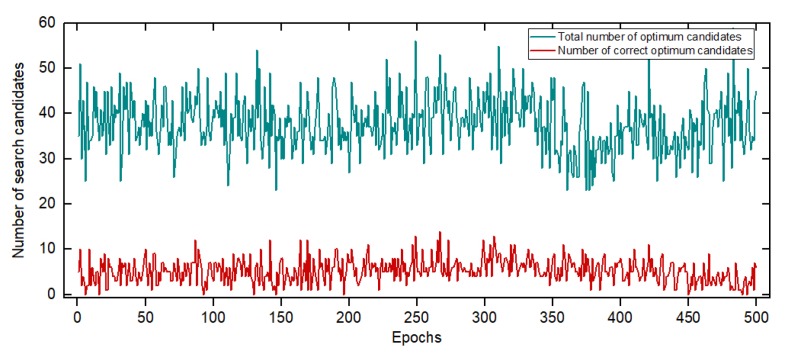
Number of optimum candidates searched in x,y,z domain.

In the 3D scene test, the prior point of each epoch used the EKF result, and the other parameters were settled the same as the above test. The number of optimum candidates in each epoch is shown in Figure 11.

**Figure 12:**
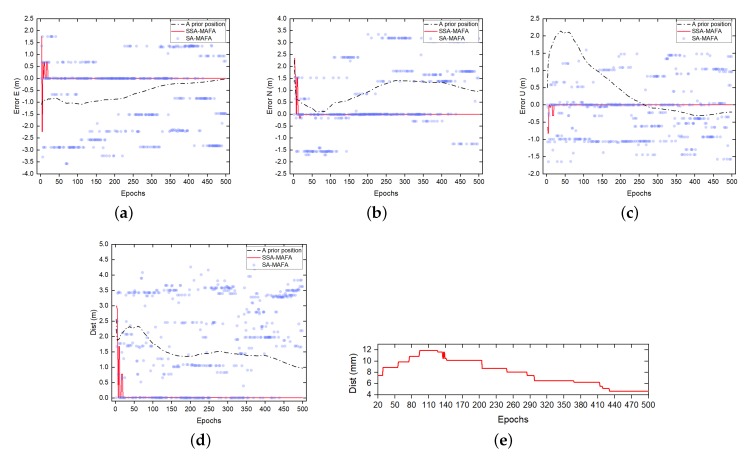
The error results (in meters) of searching in x,y,z domain by using SSA-MAFA and SA-MAFA. (**a**) The errors in East direction. (**b**) The errors in North direction. (**c**) The errors in Up direction. (**d**) The errors in both East, North and Up direction, where Dist = $\sqrt{err_{E}^{2}+err_{N}^{2}+err_{U}^{2}}$. (**e**) The errors of the convergence result by using SSA-MAFA method from epoch 20 to epoch 500.

Since the search procedure was carried out on multiple layers in this test, the total number of optimum candidates increased, while the failure rate of obtaining the correct optimum candidates decreased. The reason for less failure rate of obtaining the correct candidates is that the length between each search layer we choose is efficient enough, making search points in more layers fall into the correct Voronoi Cell. The error result of SSA-MAFA compared with the SA-MAFA method at different epochs is shown in Figure 12.

It is shown that the SSA-MAFA method can converge to the correct solution after handling nineteen epochs of data. In contrast, the SA-MAFA method may fail to give out the correct solution owing to the existence of a false global optimum. However, we must admit that the vast search region settled in our test is not that accurate, which may bring the false global optimum points with a certain probability. However, our method can be very robust in this situation, even if the accuracy of the prior position is down to several meters away from the referenced value.

### 3.2. Search Efficiency of SSA-MAFA

The SSA-MAFA method is a multi-layer search algorithm. Thus the efficiency of the search in one layer is essential. The search speed is mainly control by the factor—decreasing coefficient *d* and the number of inner loops NIL. The search efficiency in one search layer under different values of *d* and NIL were tested, and the result is shown in Table 1.

With the decreasing of the parameters value *d* and NIL, the search speed can be much quicker. However, the search for the correct optimum points can be less accurate, which leads to an increase in the number of epochs took for convergence to the correct solution, shown in Table 2.

On the other hand, the search time is of only one search layer, so the number of search layers determines the total search time. Taken for an example, if search width in *z* direction is ±0.5 m, the number of search layers is 1/0.08=13, the total search time is about 0.668 s and 0.156 s with parameters settled as d=0.9, NIL=80 and d=0.8, NIL=20, respectively. The search time results are all much slower than the LAMBDA method, but it is a huge improvement over the traditional AFM method. Nevertheless, with parameter *d* settled down to 0.8, there is a chance for the failure to obtain the correct solution. Thus, it is a trade-off between search speed and search accuracy. As the search efficiency for static positioning is not that important, the number of epochs taken makes the deal, and the parameter can then be settled as d=0.9, NIL=100. In the kinematic positioning, the search efficiency becomes significantly important, parameters d=0.9, NIL=50 can then be used considering both efficiency and accuracy. Meanwhile, the search time should be understood as only a conservative result from the preliminary test, owing to the non-interference of the search on different layers, the optimization of code program can ultimately achieve a parallel search and reduce the search time significantly, which is what we need to do in the future.

## 4. Conclusions

The solution of carrier phase ambiguity is essential for precise GNSS positioning. Methods of searching in the coordinate domain show their advantage over the methods based on ambiguity fixing, for example, immune to cycle slips, far fewer epochs taken for obtaining the correct solution. The drawbacks of low computation efficiency and the existence of the false global optimum points make the AFM class methods unfriendly for the single-frequency receivers.

The new approach derived in this paper is based on the MAFA method and focused on obtaining an accurate solution by using the single-frequency receivers without solving the ambiguity fixing problem. The mathematical model expressed by some formulas was presented. The algorithm of the whole computational process has been proposed. As shown in the experiment section, the proposed method can obtain the correct solution by handling only two epochs of data when the *z* coordinate fixed to the referenced value. Meanwhile, the result of searching in x,y,z domain can converge to the correct solution, a millimeter level of accuracy, dealing with nineteen epochs of data. Even though the set up of the search region is large, and the prior position is several meters away from its referenced value, the new approach shows its robustness compared with the SA-MAFA method, and far superior to the traditional AFM algorithm.

The search time of the new approach is another matter, mainly dependent on the number of search layers in *z* direction, that is to say, the more accuracy of the prior *z* coordinate value, the more efficient our method is. As the SSA-MAFA method is a multi-layer search algorithm, owing to the non-interference of the search on different layers, we believe the search time can be decreased significantly by using code program optimization to achieve a parallel search. We plan to continue the research on this problem and apply the SSA-MAFA method to achieve a real-time, accurate kinematic positioning.

## Figures and Tables

**Table 1:** Total Search time of SSA-MAFA (in seconds) for one search layer, where *d*, decreasing coefficient of the standard jump amplitude; NIP, number of inner loops for the search procedure with one standard jump amplitude.

	NIL	100	80	50	20
*d*					
0.99		1.076	0.862	0.568	0.226
0.9		0.112	0.086	0.0535	0.021
0.8		0.053	0.041	0.026	0.012

**Table 2:** Epochs took for convergence to the correct solution with a data sampled rate 5 Hz, where *d*, decreasing coefficient of the standard jump amplitude; NIP, number of inner loops for the search procedure with one standard jump amplitude.

	NIL	100	80	50	20
*d*					
0.99		2	7	4	6
0.9		2	6	19	11
0.8		10	24	33	27

## Abbreviations

The following abbreviations are used in this manuscript:

GNSS	Global Navigation Satellite System
AFM	Ambiguity Function Method
MAFA	Modified Ambiguity Function Approach
SA-MAFA	Simulated Annealing Modified Ambiguity Function Approach
SSA-MAFA	Segmented Simulated Annealing Modified Ambiguity Function Approach
MAFV	Modified Ambiguity Function Value
LAMBDA	Least-squares Ambiguity Decorrelation Adjustment
LSA	Least Squares Adjustment
DD	Double Differential
MIRLS	Mixed Integer-Real Least Squares
KDE	Kernel Density Estimation
NIL	Number of the Inner Loop
PDF	Probability Density Function
